# Remedy for Toothache

**Published:** 1868-12

**Authors:** 


REMEDY FOR TOOTHACHE.—Alum, reduced to an impalpable powder, two drahms; nitrous spirits of ether, seven drahms; mix and apply to the tooth.

